# Uncommon manifestation of poisoning with a mixture of pesticides

**DOI:** 10.1002/ccr3.5365

**Published:** 2022-02-07

**Authors:** Shafeajafar Zoofaghari, Navid Namakizadeh Esfahani, Amirhossein Akhavan Sigari, Nasim Tavakoli, Mozhdeh Hashemzadeh, Nastaran Eizadi‐Mood

**Affiliations:** ^1^ 48455 Isfahan Clinical Toxicology Research Center Department of Clinical Toxicology Isfahan University of Medical Sciences Isfahan Iran; ^2^ 48455 School of Medicine Isfahan University of Medical Sciences Isfahan Iran; ^3^ 48455 Clinical Informationist Research Group Health Information Research Center Isfahan University of Medical Sciences Isfahan Iran

**Keywords:** chlorpyrifos, diazinon, pyrethrins

## Abstract

The ingestion of pesticides for suicide commitment purposes is common in developing countries. We present a case of suicide with ingestion of mixed pesticides. The autopsy findings showed the presence of diazinon, chlorpyrifos, trifluralin, fenpropathrin, pyriproxyfen, and cypermethrin in his body. Clinicians managing poisoning cases need to be aware of poisoning with mixture of pesticides as a rare but highly fatal outcome.

## Graphical abstract

The use of pesticides is one of the most common methods of suicide in developing countries. Management and treatment are difficult in patients who use a combination of pesticides, because each poison has its own different and unique side effects.

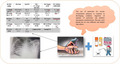

## 1 INTRODUCTION

Organophosphates (OP), carbamates, pyrethroids, nitrophenols, organochlorines (OC), and chlorophenols are the most common pesticides causing accidental and intentional poisoning.^1^, ^2^, ^3^, ^4^ The use of these products for suicide commitment purposes, especially in developing countries, is a major health problem.^5^, ^6^, ^7^, ^8^ In Iran, 19% of poisoning is related to OP and similar compounds.^9^ Cholinesterase inhibitors including organophosphate compounds (OPCs) and carbamates may be rapidly absorbed via dermal, conjunctive, respiratory, and gastrointestinal routes.^10^ Inhibition of acetylcholinesterase leads to the accumulation of acetylcholine at the terminal endings of all postganglionic nerves and neuromuscular junctions. Clinical manifestations of OPCs poisoning are due to muscarinic, nicotinic, and central nervous system (CNS) receptor overstimulation.^11^ Carbamates poorly pass the blood‐brain barrier and have less CNS effects than organophosphates.^12^

Pyrethroids are synthetic derivatives of natural pyrethrins. Pyrethroids enter the body through skin, inhalation, and food/water ingestion.^13^ There are two classical presentations in pyrethroids poisoning. Type I syndrome is characterized by tremors, and type II pyrethroids are characterized by choreoathetosis and salivation.^14^

Nitrophenols are among the most important industrial organic compounds. Acute inhalation or ingestion of nitrophenols in humans causes headaches, drowsiness, nausea, and cyanosis.^15^ Organochlorine pesticides are synthetic pesticides that are known for their high toxicity, slow degradation, and bioaccumulation.^16^ Seizure, tachycardia, and arrhythmia may be their most important clinical manifestations. Also, coma and respiratory depression may follow the seizures.^17^

Chlorophenol compounds are ubiquitous contaminants in the environment. Chlorophenols are known to be harmful toxic substance, because they easily penetrate skin and epithelium, leading to damage and necrosis.^16^ High exposure can cause headache, dizziness, tremors, seizures, coma, and even death. 4‐Chlorophenol may damage the liver and kidneys.^18^

Ingestion of mixed pesticides may produce different clinical manifestations due to their different effects on body systems. The literature review did not show any human poisoning using combination of these above‐mentioned six pesticides. Here, we report case of suicide commitment using fenpropathrin poison that resulted in death. The results of the autopsy of this case showed the presence of a combination of diazinon, chlorpyrifos, trifluralin, fenpropathrin, pyriproxyfen, and cypermethrin.

## 2 CASE HISTORY

**FIGURE 1:**
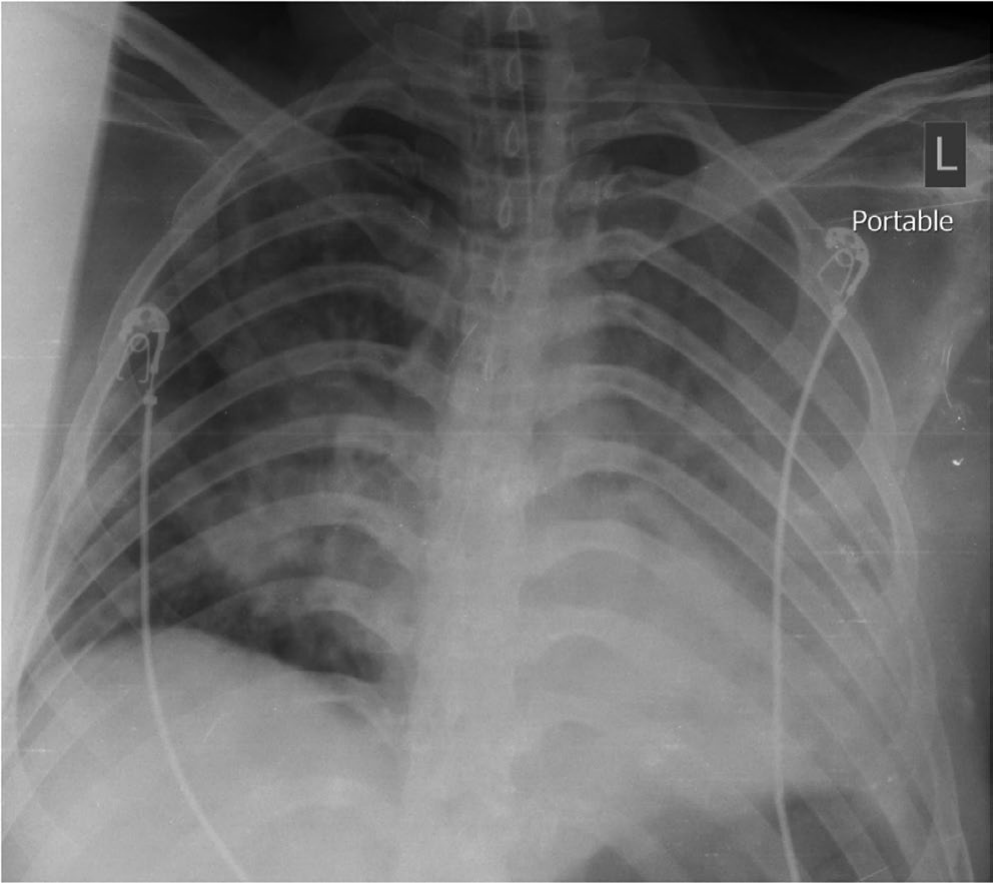
Chest X‐ray

A 26‐year‐old man was referred to the emergency department, with a decreased level of consciousness, bloody discharge in the nasogastric tube, and dark brown urine in the urinary bag. After triage, the patient was immediately transferred to the poisoning intensive care unit (ICU), intubated, and cared for with mechanical ventilation. According to the history of the companions, the patient took an unknown amount of fenpropathrin solution 6 h before hospital admission in order to commit suicide. The patient had a history of suicide, hanging in the last year, and seizures. Examinations during triage reported a low level of consciousness (stupor/coma), fixed mydriasis, nystagmus, inward iris deviation, and distal filiform pulses. The vital signs at admission have been shown in Table 1. Respiratory and metabolic acidosis, leukocytosis, acute kidney injury, and liver function test abnormality were the important laboratory findings. The patient's blood was clotting at the time of blood sampling and also in laboratory tubes. The results of the patient's clinical examinations and laboratory tests during triage are presented in Table 2. Patient's chest X‐ray is also shown in Figure 1. Bronchial lavage and culture colony count showed no bacteria growth after 48 h.

**TABLE 1:** Results of clinical examination and laboratory tests during triage

BP: 130/65 (mmHg)	PR: 115/min	O_2_Sat: 95%	WBC: 21.7 × 10^3^/mm^3^ (4000–10,000)	Hb: 17.3 gm/dl (14–18)	PLT: 241,000/ml (150,000–450,000)
Cr: 2.04 mg/dl (0.86–1.2)	LDH: 3875units/L (50–150)	INR: 2.29 (1–1.2)	PT:22.9 s (10–12)	Na:148 mEq/L (136–145)	K: 4.2 mEq/L (3.5–5.1)
ESR:2 mm/h Male: (1–13)	CRP:2 mg/dl (0–1)	VBG:pH:6.90 (7.32–7.43)	PCO_2_: 122.3 mm Hg (41–51)	HCO_3_:17.9 (23–29)	BEecf: −15
Base excess: −11.8	BB: 33.4	PO2: 41.1 mm Hg (30–40)	ALT: 30 units/L (1–21)	AST:143 units/L (7–27)	Blood group and Rh: O^+^
BUN:17 mg/dl (8.8–20)	Hematocrit: 44.4% (42–52)	BS:231 mg/dl (70–100)	ALP:249 units/L (50–160)	RBC: 5.6 × 10^6^/mm^3^ (3.9–5.9)	D‐Dimer: 10,000<μg/L (<500)
PTT: 62.1 s (28–40)	RDW: 15.2% (11–13)	MCV: 91 cu µm (80–100)	Lymphocyte: 18.8%	Neutrophil: 79%	

Abbreviations: ALP, alkaline phosphatase; BP, Blood pressure; BUN, blood urea nitrogen; Cr, creatinine; CRP, C‐reactive protein; Hb, hemoglobin; INR, international normal ratio; K, serum potassium; LDH, lactate dehydrogenase; MCV, mean corpuscular volume; Na, serum sodium; PLT, platelet; PR, pulse rate; RBC, red blood cell; WBC, white blood cell.

**TABLE 2:** Results of clinical examination and laboratory tests during triage

BP = 130/65	PR = 115/min	O_2_sat = 95%	WBC = 21,700	HB = 16/8	PLT = 241,000
Crea = 2/01	LDH = 3872	INR = 2/29	PTT = 22/9	Na = 148	K = 4/2
ESR = 2	CRP = 2	VBG:PH = 6/90	PCo2 = 122/3	Hco3 = 17/9	BEecf = −15
BE = −11/8	BB = 33/4	Po2 = 61/1			

Patients received sodium bicarbonate STAT (1–2 mEq/kg), pantoprazole (40 mg twice a day, intravenously [IV]), vitamin C IV infusion (150 mg/h), and four units of fresh‐frozen plasma (FFP) during hospitalization. Two units of pack cell were also reserved. Sodium dithionate test for paraquat poisoning was negative. Double lumen urinary catheter was implanted, and after rinsing the bladder, the patient's urine color became clearer. Due to the critical condition of the patient, it was not possible to perform a brain CT scan for him. However, internal, heart, anesthesia, surgery, and urology consultations were performed. Echocardiography showed 30% left ventricular ejection fraction (LVEF = 30%). During his hospitalization period, the patient suffered from hypotension (systolic blood pressure <90 mmHg), decreased oxygen saturation, and heart rate (BP = 102/56 mmHg, O_2_ saturation = 51%, and pulse rate = 54/min). Infusion of dobutamine (5–20 μg/kg/min), norepinephrine (0.1–1 μg/kg/min), hydrocortisone (100 mg IV bolus and 100 mg four times a day), intralipid 10% (100 ml infusion during 1 h), ceftriaxone (2 gram IV stat infusion within 30 min and one gram infusion for every 12 h), and clindamycin (600 mg IV infusion within 1 h every 8 h) were administered. Setting of ventilator was adjusted by intensive care unit specialist in the hospital.

Although hemodialysis was suggested by internal medicine specialist, the patient developed cardiac arrest before performing it. Despite resuscitation, the patient died about 8 h after admission. Finally, the patient's body was autopsied and the toxicological results showed the presence of diazinon, chlorpyrifos, trifluralin, fenpropathrin, pyriproxyfen, and cypermethrin in his blood. The results of histopathology autopsy could not be provided.

## 3 DISCUSSION

The case presented in this study involved a unique scenario of multiple toxin ingestion, which imposed diagnostic and therapeutic difficulties. The patient was found to have poisoning with multiple pesticides. Fenpropathrin is a synthetic pesticide that belongs to the class of pyrethroids, which are neurotoxic and can cause degenerations of the dopaminergic neurons of the central nervous system.^19^, ^20^ Other pesticides like organophosphates are also neurotoxic, and in case of poisoning, these can present with a range of muscarinic, nicotinic, and CNS symptoms.^20^, ^21^, ^22^ Moreover, trifluralin is an approved commercial herbicide, which is practically not acutely toxic. However, solvents used in trifluralin may irritate the eyes and skin in human beings. In addition, its accumulation in both soil and water over time has been proposed to be toxic. Therefore, the acute ingestion of trifluralin in poisoned patients shall not be an area of concern.^23^, ^24^

There are few reports on multiple toxin ingestions in the current literature, hindering the management and rapid diagnosis in such patients. Mishra et al. in their study reported a 27‐year‐old male case who had ingested a compound mixture of dichlorvos and profenofos. The patient developed nausea and vomiting and had a Glasgow Coma Scale (GCS) of 3/15 immediately upon his arrival to the emergency department. Arterial blood gas measurements of the patient revealed severe metabolic acidosis with a pH of 7.03. As well, by passing 2 days from the admission, the patient died.^25^ The poor outcome in the patient after ingestion of compound organophosphates could probably be due to his low mental status and the significant acidosis. Our patient was also similar to that case in matters of consciousness levels and pH measurements (pH = 6.9).

In an observational study conducted on 90 patients with either chlorpyrifos, cypermethrin, or a mixed poisoning of these two, none of the patients with isolated poisoning died. On the other hand, four out of 32 patients with mixed chlorpyrifos and cypermethrin, died, further emphasizing the importance of multiple toxic ingestion in a patient.^23^

Tripathi et al. in their study presented a series of eight cases over a 1‐year period with intentional ingestion of a pyrethroids and organophosphate compound. All these patients were comatose upon the presentation to the emergency department and suffered from respiratory distress. One patient died due to severe aspiration of toxins after vomiting, and other seven patients were managed to be completely recovered after proper ICU care.^1^ There have been reports of mixed poisonings, where anticholinesterase agents such as organophosphates and anticholinesterase carbamates have been mixed with organochlorines. In such cases, the cholinergic symptoms may be prominent on presentation, but aggressive treatment of the cholinergic findings leaves the subject with the symptoms of organochlorines poisoning, which needs additional treatment. Also, mixed poisoning of pyrethroids with organophosphorus compounds is associated with significant toxicity and death.^14^

Although reports are rare in this regard, patients with compound toxic ingestion have poor outcomes and all of them are presented to the hospital with altered mental status, which makes proper history taking difficult and may consequently delay the diagnosis. The above‐mentioned case ingested a mixture of pesticides and died after nearly 12 h from his admission because of the multiorgan failure. Medical providers should be alert to the possibility of mixed poisonings in the diagnosis and management of pesticide poisonings.^26^, ^27^ We could not measure the serum levels of toxins as it was not available in our hospital, which could be a limitation of our study.

## AUTHOR CONTRIBUTIONS

Shafeajafar Zoofaghari contributed to monitoring the accuracy of medical content, manuscript preparation, and editing. Navid Namakizadeh Esfahani contributed to literature search, data acquisition, and manuscript preparation. Amirhossein Akhavan Sigari contributed to literature search, data acquisition, and manuscript preparation. Nasim Tavakoli contributed to monitoring the accuracy of medical content. Mozhdeh Hashemzadeh contributed to manuscript preparation, editing, and review Email: mojdeh.hashemzadeh@gmail.com . Nastaran Eizadi‐Mood contributed to literature search and manuscript editing. All authors have approved the revised version; AND has agreed to be personally accountable for the author's own contributions and to ensure that questions related to the accuracy or integrity of any part of the work, even ones in which the author was not personally involved, are appropriately investigated, resolved, and the resolution documented in the literature.

## ETHICAL APPROVAL

This report has been approved by the ethics committee of Isfahan University of Medical Sciences (Ethical Number: IR.ARI.MUI.REC.1400.023).

## CONSENT

The authors certify that written consent has been obtained from the patient.

## Data Availability

The data are available from the corresponding author upon reasonable request.

## References

[1] Tripathi M , Pandey R , Ambesh SP , Pandey M . A mixture of organophosphate and pyrethroid intoxication requiring intensive care unit admission: a diagnostic dilemma and therapeutic approach. Anesth Analg. 2006;103(2):410‐412. 16861425 10.1213/01.ane.0000222470.89210.5a

[2] Raina S , Mahesh DM , Vivek S , Kaushal S , Dalip G . Self injection of dichlorvos, an organophosphorus compound. Online J Health Allied Sci. 2008;7(2):9.

[3] Organophosphate and carbamate poisoning [Internet] . 2020. https://www.lib.utdo.ir/contents/organophosphate‐and‐carbamate‐poisoning?search=organophosphate%20poisoning&source=search_result&selectedTitle=1~28&usage_type=default&display_rank=1. Accessed January 25, 2021.

[4] Ramchandra AM , Chacko B , Victor PJ . Pyrethroid poisoning. Indian J Crit Care Med. 2019;23(4):267‐271. 10.5005/jp-journals-10071-23304 PMC6996658 32021002

[5] Aardema H , Meertens JH , Ligtenberg JJ , Peters‐Polman OM , Tulleken JE , Zijlstra JG . Organophosphorus pesticide poisoning: cases and developments. Netherlands J Med. 2008;66(4):149‐153. 18424861

[6] Lima JS , Reis CAG . Poisoning due to illegal use of carbamates as a rodenticide in Rio De Janeiro. J Toxicol Clin Toxicol. 1995;33(6):687‐690. 8523493 10.3109/15563659509010629

[7] Boumba VA , Rallis GN , Vougiouklakis T . Poisoning suicide with ingestion of the pyrethroids alpha‐cypermethrin and deltamethrin and the antidepressant mirtazapine: a case report. Forensic Sci Int. 2017;274:75‐78. 27899216 10.1016/j.forsciint.2016.11.023

[8] Cha YS , Kim H , Cho NH , et al. Pyrethroid poisoning: features and predictors of atypical presentations. Emerg Med J. 2014;31(11):899. 23959805 10.1136/emermed-2013-202908

[9] Sadeghiehahari S , Farzaneh E , Amani F , Azari SH . Epidemiology of poisoning due to agricultural pesticides in patients referred to Ardabil city hospitals, 2012. J Health Hygiene. 2014;5(3):240‐247.

[10] Kecik Y , Yorukoglu D , Saygin B , Sekerci S . A case of acute poisoning due to organophosphate insecticide. Anaesthesia. 1993;48(2):141‐143. 8460762 10.1111/j.1365-2044.1993.tb06854.x

[11] Yurumez Y , Durukan P , Yavuz Y , et al. Acute organophosphate poisoning in university hospital emergency room patients. Intern Med. 2007;46(13):965‐969. 17603234 10.2169/internalmedicine.46.6304

[12] Silberman J & Alan T Carbamate toxicity. 2021. https://www.ncbi.nlm.nih.gov/books/NBK482183/. Accessed July 25, 2021.

[13] Chrustek A , Hołyńska‐Iwan I , Dziembowska I , et al. Current research on the safety of pyrethroids used as insecticides. Medicina (Kaunas). 2018;54(4):61. 10.3390/medicina54040061 PMC6174339 30344292

[14] Ramchandra AM , Chacko B , Victor PJ . Pyrethroid poisoning. Indian J Crit Care Med. 2019;23(Suppl 4):S267‐S271. doi:10.5005/jp-journals-10071-23304 32021002 PMC6996658

[15] Uberoi V , Bhattacharya SK . Toxicity and degradability of nitrophenols in anaerobic systems. Water Environ Res. 1997;69(2):146‐156.

[16] Jayaraj R , Megha P , Sreedev P . Organochlorine pesticides, their toxic effects on living organisms and their fate in the environment. Interdiscip Toxicol. 2016;9(3‐4):90‐100. 28652852 10.1515/intox-2016-0012 PMC5464684

[17] Igbinosa EO , Odjadjare EE , Chigor VN , et al. Toxicological profile of chlorophenols and their derivatives in the environment: the public health perspective. Scientific World J. 2013;2013:460215. 10.1155/2013/460215 PMC3649668 23690744

[18] Xiong J , Zhang X , Huang J , et al. Fenpropathrin, a widely used pesticide, causes dopaminergic degeneration. Mol Neurobiol. 2016;53(2):995‐1008. 25575680 10.1007/s12035-014-9057-2 PMC5333774

[19] Jaremek M , Nieradko‐Iwanicka B . The effect of subacute poisoning with fenpropathrin on mice kidney function and the level of interleukin 1β and tumor necrosis factor α. Mol Biol Rep. 2020;47(6):4861‐4865. 32385770 10.1007/s11033-020-05480-w PMC7295845

[20] Bradberry SM , Cage SA , Proudfoot AT , Vale JA . Poisoning due to pyrethroids. Toxicol Rev. 2005;24(2):93‐106. 16180929 10.2165/00139709-200524020-00003

[21] Goel A , Aggarwal P . Pesticide poisoning. Natl Med J India. 2007;20(4):182. 18085124

[22] Organophosphate Toxicity Medication [Internet] . 2020. https://emedicine.medscape.com/article/167726‐medication. Accessed January 25, 2021.

[23] Iyyadurai R , Peter JV , Immanuel S , et al. Organophosphate‐pyrethroid combination pesticides may be associated with increased toxicity in human poisoning compared to either pesticide alone. Clin Toxicol. 2014;52(5):538‐541. 10.3109/15563650.2014.909933 24779865

[24] Ebert E , Leist KH , Hack R , Ehling G . Toxicology and hazard potential of trifluralin. Food Chem Toxicol. 1992;30(12):1031‐1044. 1473797 10.1016/0278-6915(92)90114-z

[25] Mishra A , Pandya HV , Dave N , Mehta M . Multi‐organ dysfunction syndrome with dual organophosphate pesticides poisoning. Toxicol Int. 2013;20(3):275. 24403738 10.4103/0971-6580.121682 PMC3877496

[26] Cable GG , Doherty S . Acute carbamate and organochlorine toxicity causing convulsions in an agricultural pilot: a case report. Aviat Space Environ Med. 1999;70(1):68‐72. 9895024

[27] Thunga G , Sam KG , Khera K , Xavier V , Verma M . Profile of acute mixed organophosphorus poisoning. Am J Emerg Med. 2009;27(5):628 e621‐623. 10.1016/j.ajem.2008.08.030 19497478

